# Adaptation of the 2015 American College of Rheumatology treatment guideline for rheumatoid arthritis for the Eastern Mediterranean Region: an exemplar of the GRADE Adolopment

**DOI:** 10.1186/s12955-017-0754-1

**Published:** 2017-09-21

**Authors:** Andrea Darzi, Manale Harfouche, Thurayya Arayssi, Samar Alemadi, Khaled A. Alnaqbi, Humeira Badsha, Farida Al Balushi, Bassel Elzorkany, Hussein Halabi, Mohammed Hamoudeh, Wissam Hazer, Basel Masri, Mohammed A. Omair, Imad Uthman, Nelly Ziade, Jasvinder A. Singh, Robin Christiansen, Peter Tugwell, Holger J. Schünemann, Elie A. Akl

**Affiliations:** 1 AUB GRADE Center, Clinical Research Institute, American University of Beirut, PO Box 11-0236, Riad El Solh, Beirut, 1107 2020 Lebanon; 2 Weill Cornell Medicine-Qatar- Department of Internal Medicine, Doha, Qatar; 3 Department of Rheumatology, Medical Institute, Al Ain Hospital, Al Ain, United Arab Emirates; 4 Dr. Humeira Badsha Medical Center, Rheumatologist City Hospital, Rheumatologist Neurospinal Hospital, Dubai, United Arab Emirates; 5 Ministry of Health, Muscat Governorate, Oman; 6 Department of Rheumatology, Cairo University, Giza, Egypt; 7 Rheumatology Division, Department of Internal Medicine, King Faisal Specialist Hospital and Research Center, Jeddah, Saudi Arabia; 8 Hamad Medical Corporation, Doha, Qatar; 9 Aspetar, Doha, Qatar; 10 Jordan Hospital, Amman, Jordan; 11 Division of Rheumatology, Department of Medicine, King Saud University, Riyadh, Saudi Arabia; 12 American University of Beirut, Beirut, Lebanon; 13 Faculty of Medicine, Univeristé Saint Joseph, Beirut, Lebanon; 14 Medicine Service and Center for Surgical Medical Acute care Research and Transitions, VA Medical Center, 510, 20th Street South, FOT 805B, Birmingham, AL USA; 15 Department of Medicine at School of Medicine, and Division of Epidemiology at School of Public Health, University of Alabama, 1720 Second Ave. South, Birmingham, AL 35294-0022 USA; 16 Musculoskeletal Statistics Unit, The Parker Institute, Bispebjerg and Frederiksberg Hospital, Copenhagen, Denmark; 17 Department of Medicine, University of Ottawa, Ottawa, Canada; 18 Department of Medicine, McMaster University, Hamilton, ON Canada; 19 Department of Health Research Methods, Evidence, and Impact (HE&I), McMaster University, Hamilton, ON Canada; 20 Department of Internal Medicine, American University of Beirut, PO Box 11-0236, Riad El Solh, Beirut, 1107 2020 Lebanon

**Keywords:** Practice guideline, Adaptation, GRADE, Evidence-based medicine, Eastern Mediterranean Region, Rheumatoid arthritis, Conflicts of interest

## Abstract

**Background:**

It has been hypothesized that adaptation of health practice guidelines to the local setting is expected to improve their uptake and implementation while cutting on required resources. We recently adapted the published American College of Rheumatology (ACR) Rheumatoid Arthritis (RA) treatment guideline to the Eastern Mediterranean Region (EMR). The objective of this paper is to describe the process used for the adaptation of the 2015 ACR guideline on the treatment of RA for the EMR.

**Methods:**

We used the GRADE-Adolopment methodology for the guideline adaptation process. We describe in detail how adolopment enhanced the efficiency of the following steps of the guideline adaptation process: (1) groups and roles, (2) selecting guideline topics, (3) identifying and training guideline panelists, (4) prioritizing questions and outcomes, (5) identifying, updating or conducting systematic reviews, (6) preparing GRADE evidence tables and EtD frameworks, (7) formulating and grading strength of recommendations, (8) using the GRADEpro-GDT software.

**Results:**

The adolopment process took 6 months from January to June 2016 with a project coordinator dedicating 40% of her time, and the two co-chairs dedicating 5% and 10% of their times respectively. In addition, a research assistant worked 60% of her time over the last 3 months of the project. We held our face-to-face panel meeting in Qatar. Our literature update included five newly published trials. The certainty of the evidence of three of the eight recommendations changed: one from moderate to very low and two from low to very low. The factors that justified a very low certainty of the evidence in the three recommendations were: serious risk of bias and very serious imprecision. The strength of five of the recommendations changed from strong to conditional. The factors that justified the conditional strength of these 5 recommendations were: cost (*n* = 5 [100%]), impact on health equities (*n* = 4 [80%]), the balance of benefits and harms (*n* = 1 [20%]) and acceptability (n = 1 [20%]).

**Conclusion:**

This project confirmed the feasibility of GRADE-Adolopment. It also highlighted the value of collaboration with the organization that had originally developed the treatment guideline. We discuss the implications for both guideline adaptation and future research to advance the field.

## Background

Guidelines are considered an integral aspect of the development of a standardized high quality health care using evidence-based practices [1]. Guidelines are defined by the World Health Organization (WHO) as: “systematically developed evidence-based statements which assist providers, recipients and other stakeholders to make informed decisions about appropriate health interventions” [2].

Development of guidelines de novo faces multiple challenges including financial and human resource requirements and time constraints [3]. On the other hand, using guidelines developed for one setting in another one (i.e., guideline adoption) may be inappropriate due to differing contextual factors such as acceptability or feasibility of the proposed intervention.

Adaptation of guidelines would address the above challenges and limitations, through modifying the recommendations to account for contextual factors. It has been hypothesized that adaptation of guidelines to the local setting is expected to improve their uptake and implementation [4]. One of the challenges of the process of adapting guidelines is to keep it efficient while ensuring it is evidence-based.

A recently published  survey identified eight methods for guidelines adaptation, one of which is GRADE- Adolopment [5]. Adolopment combines the advantages of adoption, adaptation and de novo guideline development and is based on three cornerstones: (1) identifying and prioritizing credible existing guidelines or evidence syntheses that are of both interest and relevance; (2) evaluating and completing the GRADE Evidence to Decision (EtD) frameworks for each of the recommendations [6]; and (3) deciding on a final adoption, adaptation or de novo development for each of the recommendations [7].

We adoloped the recently published American College of Rheumatology (ACR) Rheumatoid Arthritis (RA) treatment guideline to the Eastern Mediterranean Region [8]. This project was a collaborative effort between the Weill Cornell Medical College in Qatar, the Middle East Rheumatoid Arthritis Consortium (MERAC), and the American University of Beirut (AUB) GRADE Center.

The objective of this paper is to describe the process used for the adolopment of the 2015 ACR guideline on the treatment of RA for the Eastern Mediterranean Region. We report on the specific recommendations resulting from this process in a separate paper.

## Methods

We used the adolopment methodology to adapt the 2015 ACR RA treatment guideline [8] to the Eastern Mediterranean Region and based the process on the GRADE-Adolopment approach [7]. Shortly after the publication of the ACR RA guidelines, (which we refer to as ‘the source guideline’), the MERAC group identified them as a priority for adaptation. The source guideline [8] used the GRADE methodology to rate the certainty of evidence. We obtained the approval of the ACR senior director of quality for using that guideline in our project.

We structured the process for the ‘adoloped guideline’ using the Guidelines 2.0 comprehensive checklist for guideline development, which consists of 18 steps [9]. Table 1 highlights what steps were transferred from the process of the source guideline, and what steps we specifically conducted for the current process. Table 2 on the other hand presents how the guideline process matches the 18 steps outlined in the ‘Guidelines 2.0’ comprehensive checklist for guideline development. We provide below a detailed description of how adolopment enhanced the efficiency of the following steps compared to de novo guideline development:

1. Groups and roles
2. Selecting guideline topics
3. Identifying and training guideline panelists
4. Prioritizing questions and outcomes
5. Identifying, updating or conducting systematic reviews
6. Preparing GRADE evidence tables and EtD frameworks
7. Formulating and grading strength of recommendations
8. Using the GRADEPro-GDT software

**Table 1:** Composition, role, and link to source guidelines for each of the three groups involved in the guideline project

Group	Composition	Role	Link to source guideline
Guideline executive committee	One rheumatologist, and three guideline methodologists, of whom two were internists and one was a general practitioner.	Oversaw the project and provided advice on both content and process related issues	One member was a methodologist for the source guideline
Guideline coordination team	Three members of the AUB GRADE center who combined expertise in clinical medicine, public health, and systematic review and guideline methodologies.	Executed the project (data collection, evidence synthesis and presentation, and developing material), and supported the panelists them throughout the process	One member was a methodologist for the source guideline
Guideline panel	Regional and international content experts (RA management), non-academic clinicians, other healthcare professionals (a nurse), and methodologists.	Prioritized questions and outcomes, provided contextual information, advised on evidence of effectiveness of interventions, and participated in a final panel meeting	One panelist was the chair of the source guideline

**Table 2:** Adolopment process and relation with the Guidelines 2.0 checklist

Guidelines 2.0 checklist		‘Adoloped guideline’ process	
Step	Description	Transferred from ‘source guideline’ process	Conducted specifically for the ‘adoloped guideline’ process
Organization, Budget, Planning and Training	*Organization, budget, planning and training* involves laying out a general but detailed plan describing what is feasible, how it will be achieved and what resources are required to produce and use the guideline. The plan should refer to a specific time period, and be expressed in formal, measurable terms.		1. Detailed plan set between the Weill Cornell Medical College in Qatar, the Middle East Rheumatoid Arthritis Consortium (MERAC), and the American University of Beirut GRADE Center 2. Funding through the Qatar National Research Fund and Weill Cornell Medical College 3. Training of panelists on the GRADE approach through training videos and a one-day workshop preceding the panel meeting
Priority Setting	*Priority setting* is the identification, balancing and ranking of priorities by stakeholders. It ensures that resources and attention are devoted to those general areas (e.g. chronic obstructive pulmonary disease, diabetes, cardiovascular disease, cancer, prevention) where health care recommendations will provide the greatest benefit to the population, a jurisdiction, or a country. A priority-setting approach needs to contribute to future plans while responding to existing potentially difficult circumstances.^92,93^	Project triggered by opportunity presented by the publication of the ACR RA guidelines.	Priority set based informal discussions with regional experts
Guideline Group Membership	*Guideline group membership* defines who is involved, in what capacity, and how the members are selected for the guideline development and at other steps of the guideline enterprise.		Regional and international content experts and methodologists recruited: into (1) the guideline executive committee; (2) the guideline coordination team; and (3) the guideline panel
Establishing Guideline Group Processes	*Establishing guideline group processes* defines the steps to be followed, how those involved will interact, and how decisions will be made.		1. Executive committee: oversaw work done and advised on process and methodology 2. Coordination team: conducted literature reviews, produced documents, and provided guidance and support to panelists 3. Panel members: were involved in prioritizing questions, contributing contextual information and participating in the final meeting 4. Decisions reached through discussion, consensus building and voting
Identifying Target Audience and Topic Selection	*Identifying target audience* involves describing the potential users or consumers of the guideline. *Topic selection* defines the topics to be covered in the guideline (e.g. diagnosis of chronic obstructive pulmonary disease).	Target audience: same as that of ACR RA guidelines, i.e., clinicians and their patients with RA. The scope (treatment of RA) and range of topics were those of the ACR RA guidelines	The executive committee selected one out of the four topics addressed in ACR RA guidelines, that is the treatment of early RA patients
Consumer and Stakeholder Involvement	*Consumer and stakeholder involvement* describes how relevant people or groups who are not necessarily members of the panel but affected by the guideline, e.g. as target audience or users, will be engaged.		The executive committee involved a rheumatology clinic nurse as panelist
Conflict of Interest Considerations	*Conflict of interest considerations* focus on defining and managing potential divergence between an individual’s interests and his or her professional obligations that could lead to questioning whether the actions or decisions are motivated by gain, such as financial, academic advancement, clinical revenue streams or community standing. Financial or intellectual or other relationships that may impact an individual or organization’s ability to approach a scientific question with an open mind are included.		The executive committee developed a policy document for both declaration (using the WHO COI form) and management of COI.
(PICO) Question Generation	*(PICO) Question Generation* focuses on defining key questions the recommendations should address, including the detailed population, intervention (including diagnostic tests and strategies) and outcomes that will be relevant for decision making (e.g. should test A be used, or should treatments B, C, D or E be used in chronic obstructive pulmonary disease).	The executive committee considered all PICO questions from the ACR guidelines for the selected topic (early treatment of RA patients)	The coordination team surveyed panelists to prioritize questions for the EMR setting. The top 8 questions were selected
Considering Importance of Outcomes and Interventions, Values, Preferences and Utilities	*Considering importance of outcomes and interventions, values, preferences and utilities* includes integrating in the process of developing the guidelines, how those affected by its recommendations assess the possible consequences. These include patient and carer knowledge, attitudes, expectations, moral and ethical values, and beliefs; patient goals for life and health; prior experience with the intervention and the condition; symptom experience (for example breathlessness, pain, dyspnea, weight loss); preferences for and importance of desirable and undesirable outcomes; perceived impact of the condition or interventions on quality of life, well-being or satisfaction and interactions between the work of implementing the intervention, the intervention itself, and other contexts the patient may be experiencing; preferences for alternative courses of action; and preferences relating to communication content and styles, information and involvement in decision-making and care. This can be related to what in the economic literature is considered *utilities*. An intervention itself can be considered a consequence of a recommendation (e.g. the burden of taking a medication or undergoing surgery) and a level of importance or value is associated with that.	The executive committee considered all outcomes addressed by ACR for the prioritized questions	1. The coordination team: • Sent outcome rating survey to panelists • Searched literature for studies on patients’ values and preferences, • Solicited panelists for additional studies on patients’ values and preferences
Deciding what Evidence to Include and Searching for Evidence	*Deciding what evidence to include and searching for evidence* focuses on laying out inclusion and exclusion criteria based on types of evidence (e.g., rigorous research, informally collected), study designs, characteristics of the population, interventions and comparators, and deciding how the evidence will be identified and obtained. It also includes but is not limited to evidence about values and preferences, local data and resources.	The ACR RA guideline working group shared their evidence reviews	The coordination team: 1. Searched for SRs and prioritized them based on directness, low risk of bias, and up-to-date 2. Updated searches for primary studies using same eligibility criteria used by ACR 3. Searched for resource use data using filter limiting search to EMR region 4. Searched for evidence on and values and preferences 5. Solicited panelists for additional studies on baseline risks and resource use
Summarizing Evidence and Considering Additional Information	*Summarizing evidence and considering additional information* focuses on presenting evidence in a synthetic format (e.g. tables or brief narratives) to facilitate the development and understanding of recommendations. It also involves identifying and considering additional information relevant to the question under consideration.	The ACR RA guideline working group shared their RevMan files	The coordination team: 1. Updated the RevMan files with newly identified studies 2. Solicited from panelist additional information relevant to the question under consideration
Judging Quality, Strength or Certainty of a Body of Evidence	*Judging Quality, Strength or Certainty of a body of Evidence* includes assessing the confidence one can place in the obtained evidence by transparently evaluating the obtained research (individual studies and across studies) and other evidence applying structured approaches. This may include but is not limited to evidence about baseline risk or burden of disease, the values and preferences, resource use (cost), estimates of effects, and diagnostic test accuracy.	The ACR RA guideline working group shared GRADE Pro files shared	The coordination team revised ratings in the GRADE evidence profiles when new evidence incorporated or when judged necessary.
Developing Recommendations and Determining their Strength	*Developing recommendations* focuses on integrating the factors that influence a recommendation using a structured analytic framework, and a transparent and systematic process. *Determining the strength of the recommendations* refers to judgments about how confident a guideline panel is that the implementation of a recommendation exerts more desirable than undesirable consequences.	No Evidence-to-Decision (EtD) tables were developed for the ACR RA guideline	The coordination team developed an EtD table for each question. The panel used the GRADE evidence profiles and EtD tables at the time of the meeting and decided on the direction and strength of the final recommendations.
Wording of Recommendations and of Considerations of Implementation, Feasibility and Equity	*Wording of recommendations* refers to choosing syntax and formulations that facilitate understanding and implementation of the recommendations. Such wording is connected to *considerations of implementation, feasibility and equity*, which refer to the guideline panel’s considerations about how the recommendation will be used and what impact it, may have on the factors described.		The panelists reviewed and refined the wording of the final recommendation.
Reporting and Peer Review	*Reporting refers to how a guideline will be made public (*e.g. *print, online). Peer review* refers to how the guidelines document will be reviewed and how it can be assessed (e.g. for errors), both internally and externally, prior to its publication by stakeholders who were not members of the guideline development group.		Pending
Dissemination and Implementation	*Dissemination and implementation* focuses on strategies to make relevant groups aware of the guidelines and to enhance their uptake (e.g. publications and tools such as mobile applications).		Pending
Evaluation and Use	*Evaluation and use* refers to formal and informal strategies that allow judgments about: evaluation of the guidelines as a process and product; evaluation of the use and/or uptake; and evaluation of impact and whether or not the guideline leads to improvement in patient or population health or other consequences.		Pending
Updating	*Updating* refers to how and when a guideline requires revision because of changes in the evidence or other actors that influence recommendations.		Pending

### Groups and roles

This step included identifying and recruiting individuals for the following three groups: a guideline executive committee, a guideline coordination team, and a guideline panel (see Table 1). A content expert (rheumatologist) and a guideline methodologist co-chaired the final panel meeting. They facilitated and steered the discussion, reflected on and summarized the panelists viewpoints, raised issues/concerns that could inform the decision-making process; and attempted to achieve consensus whenever possible. The methodologist co-chair did not vote while the content co-chair did.

In term of conflicts of interest (COI) rules, we asked the panel members to fill out an online COI declaration form, adopted from the World Health Organization. We will include these forms as an appendix in the adopted guideline manuscript. All panelists received a COI policy document explaining the approach to managing conflicts during the guideline process. The two panel co-chairs exercised the rule of “no strong advocacy” to minimize the effect of the COIs. Accordingly, panelists were invited to share their opinions and positions on each recommendation, while avoiding repetition or being verbally forceful. The co-chairs had the right to exclude panel members with COI from discussions or decision-making on recommendations they were conflicted on.

### Selecting guideline topics

The source guideline (the 2015 ACR RA treatment guideline) addressed four different topics: (*i*) management of patients with early RA (15 questions), (*ii*) management of patients with established RA (44 questions), (*iii*) management of patients with established RA with high risk (24 questions), and (*iv*) live vaccines in early or established RA patients (5 questions). Given the limited time and resources, the guideline executive committee chose to address a set of prioritized questions from the first topic, i.e., management of patients with early RA. While this choice was pragmatic it also aimed to produce a coherent set of questions, i.e., within the same type of patient population.

### Identifying and training guideline panelists

The executive committee recruited guideline panelists in a way to represent a diverse set of expertise. Panelists included regional and international rheumatologists, a nurse practicing in Qatar, and methodologists from AUB GRADE and the McMaster GRADE (Mac GRADE) centers. The regional rheumatologists were members of the Middle East Rheumatoid Arthritis Consortium (MERAC). MERAC is based at the Weill Cornell Medical College in Qatar and is comprised of rheumatologists from Jordan, Kingdom of Saudi Arabia, Lebanon, United Arab Emirates, and Qatar. MERAC’s research focuses on studying rheumatoid arthritis in the Middle East.

One month ahead of the panel meeting, we shared with the panelists training videos on the GRADE approach for guideline development found on the webpage of the MacGRADE center. Also, the first day of the guideline meeting consisted of a workshop on the use of the GRADE Adolopment methodology. The workshop used material related to the one of the planned guideline questions.

### Prioritizing questions and outcomes

The core team at the AUB GRADE Center developed a prioritization survey that included all questions from the source guideline addressing the prioritized topic, i.e., early RA. We asked the panelists to rate the importance of these questions on a Likert scale of 1–5 (least - most important), from the perspective of patients in the EMR. We selected 8 questions taking into consideration panelists’ ratings and the coherence amongst those questions. We communicated the list of selected questions to the panelists for approval. It was not possible afterwards to change or add questions to the project.

Then, we sent the panelists a survey to prioritize patient important outcomes relating to the selected questions. The survey specifically asked participants to “consider outcomes that might be important to someone making a decision to use or not to use the treatment”. The panelists used a Likert scale of 1–9 to rate the importance of these outcomes for making a decision as follows: (1–3) not important; (4–6) important but not critical; and (7–9) critical.

### Identifying, updating or conducting systematic reviews

**Fig. 1:**
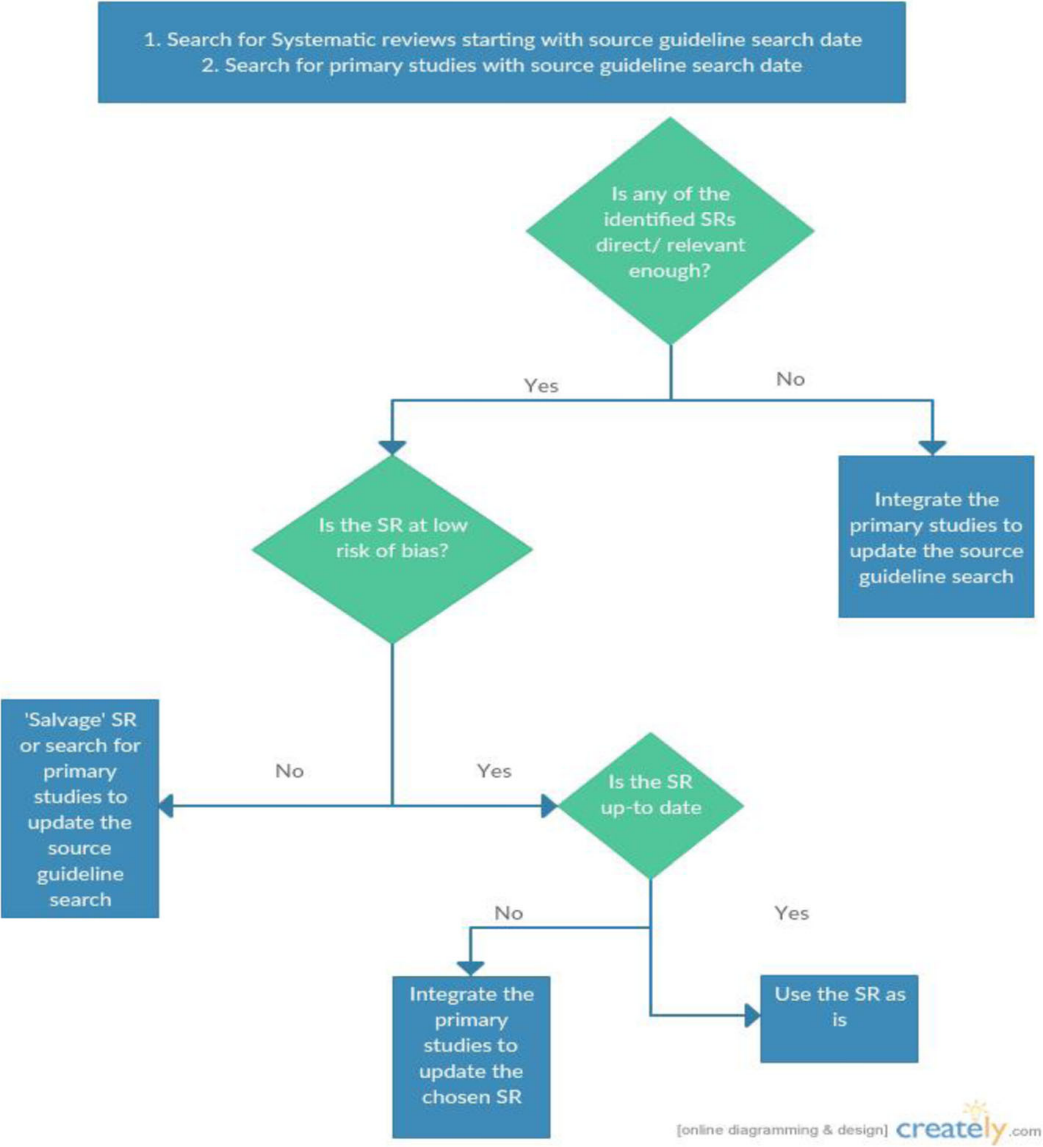
Algorithm of our search and use of the identified evidence. *Salvaging the Systematic Review would entail redoing the deficient part of the methods (e.g. meta-analysis section)

Figure 1 depicts the process of searching and using the identified evidence for the recommendation questions selected by the panel. We ran two searches for systematic reviews and primary studies respectively. We searched Medline, Embase, Cochrane and Epistemonikos electronic databases from the last search date of the source guideline in September 2014, till February 2016. We used the same search terms as the source guideline search; we only added study design filters for primary studies and systematic reviews respectively. The search terms included both medical subject headings (MeSH) and text words.

We used standards systematic review methodology including duplicate and independent approach to title and abstract screening, full text screening, and data abstraction. We conducted calibration exercises, used standardized and pilot tested forms, and relied on a third reviewer to resolve disagreements.

When evaluating the potential use of identified systematic review, we considered the following three characteristics as important:

- Relevance (directness): we assessed the relevance of identified systematic reviews by matching their PICO to the PICO of the guideline questions. The minimum requirement was for the Population, Intervention and Control elements to match to a reasonable degree, i.e., not to have serious indirectness for more than one of the three elements.
- Quality (risk of bias): we assessed the risk of bias of relevant systematic reviews using AMSTAR [10]. If we identified more than one relevant systematic review we prioritized the one with the highest quality.
- Being Up to date: we assessed whether the systematic review judged to be relevant and of highest quality was up to date. In case we had identified more than one systematic review, the judgment of relative up-to-dateness would have considered whether the systematic reviews included all relevant studies. When we identified new primary studies, we integrated the findings in the chosen systematic review.

When we identified no usable systematic review (based on the three above criteria), we updated the systematic review conducted by the source guideline-working group using the results of the search for primary studies.

In addition, we searched the literature for studies and data relevant to patients’ values and preferences and economic data. The eligibility criteria related to both the population of interest (rheumatoid arthritis) and the interventions of interest. We searched Medline using both medical subject headings (MeSH) and text words related to ‘rheumatoid arthritis’ and the interventions of interest. We used a filter-limiting search to EMR region when searching for economic data but not when searching for values and preferences. Also, we limited to a period covering the last 10 years when searching for values and preferences. In addition, we solicited panelists for additional studies on baseline risks and economic data.

### Preparing GRADE evidence tables and EtD frameworks

In preparation for the guideline panel meeting, we used GRADEpro-GDT guideline development tool (www.gradepro.org) to develop for each recommendation question the standard tables proposed by the GRADE working group to facilitate the process [11]:

1. Evidence Tables: they provide for each of the outcomes of interest a summary of the synthesized evidence (ideally based on a meta-analysis) and the rating of the certainty of the evidence. The GRADE working group has developed two versions of these evidence tables: Evidence Profiles (EP) (a more detailed version) and Summary of Findings tables (SoF) (a less detailed version) [12];
2. Evidence to Decision frameworks (EtD): they are intended to facilitate the panel’s decision-making process for going from evidence to recommendation by summarizing in a structured and transparent way the evidence for the following factors: benefits and harms, values and preferences, cost, cost effectiveness, equity, acceptability and feasibility [6, 13]. As the source guideline did not include EtD frameworks, we developed our own for this project.

The team that conducted the systematic review for the source guideline, shared with us, with the approval of ACR senior director of quality, files relevant to the recommendation questions of interest (e.g., Review Manager and GRADEpro-GDT files).

### Formulating and grading strength of recommendations

The guideline panelists revised the Evidence Profiles and provided input prior to the panel (face-to-face) meeting. During the panel meeting, we used the GRADE Evidence-to-Decision frameworks to assist the panel in formulating and grading the final recommendations. During this process, the panel decided to downgrade the evidence for three recommendations from low to very low due to a judgment for the imprecision factor that was different from that of the source guideline.

We intentionally did not expose the panelists to the recommendations from the source guideline prior to finalization of the recommendations. For each of these recommendations, we expected the use of the adolopment process to lead in one of three potential outcomes:

- Adoption of the recommendation, i.e., the use of the original recommendation as is;
- Adaptation of the recommendation, i.e., the modification of the original recommendation;
- De novo development of the recommendation, i.e., the creation of a new recommendation.

### Using the GRADEpro-GDT software (www.Gradepro.Org)

We used the GRADEpro-GDT software to conduct the following tasks:

- Collect the COI disclosures statements of panelists
- Update the evidence profiles and develop EtD frameworks for each of the guideline’s questions;
- Facilitate the panel discussions by projecting the tables in real time during the meeting;
- Export tables in word format to include in the final guideline report.

## Results

The adolopment process yielded eight recommendations, which will be detailed in another paper. Below we provide a description relating to the: (1) timeframe of the process, (2) results of the literature review, and (3) change in the certainty of evidence and the strength of recommendation.

### Timeframe

Table 3 provides a detailed description of the timeframe for the different steps of the adolopment process. The process took 6 months starting January 2016 with a project coordinator (AD) dedicating 40% of her time, and the two co-chairs (TA, EAA) dedicating 5% and 10% of their times respectively. In addition, a research assistant (MH) worked 60% of her time over the last 3 months of the project. We dedicated the first 2 months to setting up the project, including drafting the project overview, selecting and inviting the panelists, and collecting their declarations of conflicts of interest (COI). The most time consuming steps were: (1) deciding what evidence to include and searching for evidence (2) summarizing the evidence and considering additional information and (3) judging strength or certainty of a body of evidence. We completed these steps over a period of 3 months. To make the process more efficient we ran these steps in parallel. Once these steps were finalized we presented the synthesized evidence in a three-day face-to-face panel meeting in Qatar in May 2015.

**Table 3:** Timeframe for the different steps of the process (January–June 2016)

	January	January	February	February	March	March	April	April	May	May
Organization, Budget, Planning and Training	×	×								
Priority Setting	×	×								
Guideline Group Membership			×							
Establishing Guideline Group Processes			×							
Identifying Target Audience and Topic Selection				×						
Consumer and Stakeholder Involvement				×						
Conflict of Interest Considerations				×						
(PICO) Question Generation					×					
Considering Importance of Outcomes and Interventions, Values, Preferences and Utilities						×	×			
Deciding what Evidence to Include and Searching for Evidence						×	×			
Summarizing Evidence and Considering Additional Information								×	×	
Judging Quality, Strength or Certainty of a Body of Evidence								×	×	
Developing Recommendations and Determining their Strength										×
Wording of Recommendations and of Considerations of Implementation, Feasibility and Equity										×

### Results of the literature search

Table 4 describes the results of the systematic literature search per question. The search for systematic reviews of effectiveness, with no date limit, yielded 772 papers, two of which were relevant to the project [14, 15]. The search for primary studies of effectiveness done after the search date for the ACR RA treatment guideline yielded 2051 papers, five of which were eligible [16–20].

**Table 4:** The number of included studies per question resulting from different literature searches

Question number	Studies included in source guideline	SR search	Screening of SR for primary studies^a^	Primary study search
Question 1	–	1	2	1
Question 2	3	0	–	0
Question 3	4	0	–	0
Question 4	7	1	2	3
Question 5	0	0	–	2
Question 6	1	0	–	1
Question 7	1	0	–	0
Question 8	1	0	–	0

*SR* systematic review

^a^Primary studies included in the identified systematic review and not included in the source guideline search

With regards to our search for studies on patients’ values and preferences, we identified 16 relevant studies but none were specific to the Eastern Mediterranean Region context. The information we retrieved looked at outcome valuation and medication preference in general (i.e., not specific to our questions). We did not identify any studies on resource use relevant to the Eastern Mediterranean Region.

### Change in the certainty of evidence and the strength of recommendation

After we formulated the eight final recommendations, we compared the certainty and strength of each of the adoloped recommendations to corresponding recommendations from the source guideline. The certainty of the evidence of three of the eight recommendations changed: one from moderate to very low and two from low to very low. The factors that justified a very low certainty of the evidence in these three recommendations were: serious risk of bias and very serious imprecision. The strength of five out of the eight recommendations changed from strong to conditional. The factors that justified the conditional strength of these 5 recommendations were the following: cost (*n* = 5), impact on health equities (*n* = 4), the balance of benefits and harms (*n* = 1) and acceptability (n = 1).

## Discussion

We describe in this paper the process used for the adaptation of the 2015 ACR RA treatment guideline for the EMR. The process, which took 6 months was based on the GRADE-Adolopment approach and resulted in a total of 8 recommendations on the management of the early RA. The strength of five out of the eight recommendations changed from strong to conditional.

This project confirmed the feasibility of GRADE-Adolopment [7]: (1) Use of existing evidence syntheses for every recommendation question (2); short time frame for completing the guideline (about 6 months) thanks to using existing systematic reviews and collaborating with the source guideline organization as detailed below; and (3) use of a transparent and structured process in formulating the recommendation.

Our collaboration with the source guideline organization proved to be crucial for the success of the project. A major contributing factor was the willingness of the senior director of quality for the ACR to allow the unrestricted use of their recently published guideline as the basis for the adolopment process. As a result, we used ACR RA treatment guideline recommendation questions as a starting point to prioritize our questions; the participation of the chair and the methodologist of the ACR treatment guideline gave the panel unique insight into the source guideline’s panel decision-making process; it helped with clarifying any uncertainties regarding the evidence synthesis and formulation of the final recommendations. In addition, the fact that the two guideline efforts used the same methodology (i.e., GRADE), and the same the tools (e.g., RevMan, GRADEPro-GDT) made our process more efficient.

This project was not without any challenges or barriers. One major challenge was the scarcity of evidence for contextual factors such as values and preferences, and economic data. The panel relied on its members’ expert opinion to judge those factors as part of the EtD. Another challenge was the need to develop a plan (as describe above) to build the capacity of the panelists and of some members of the technical team in guideline development and adolopment. We were also challenged with the relatively short timeframe of 6 months. We managed by employing efficient processes, having the members of the core team dedicate a larger amount of their time to the project while limiting the number of questions to address.

To keep adolopment of guidelines efficient, guideline developers might need to accept a number of restrictions based on our experience:

- Accepting definitions or classifications used by the source guidelines for the condition, the interventions, and the outcomes
- Using either the exact same question or a question with narrower scope, compared to the source guidelines. Otherwise, the adapting group must run a new search strategy to ensure capturing all studies relevant to their new question
- Accepting the outcomes and the associated timeframes as prioritized by the source guidelines
- Accepting any limitations in the process of the source guidelines related to the systematic review process (e.g., search strategy, risk of bias assessment, rating of the certainty of evidence).

Having considered the above, it is also ideal that the guideline adolopment panel makes its own judgments about context sensitive aspects of the decision-making process:

- The indirectness of the evidence as it relates to the local population (i.e., the local population might be different from the population in the original setting and from participants included in trials);
- The indirectness of the evidence as it relates to the intervention (e.g., it might not be logistically possible to locally reproduce the ‘treat to target’ strategy in a way that is similar to what the relevant trials did and to what is available in the source setting);
- The baseline risk of the outcomes of interest (e.g., incidence of malignancies higher in the local population compared to the source setting)
- The values that the local population attach to the outcomes of interest (e.g., reduction in side effects) and their preferences for the interventions of interest (e.g., intravenously administered biologics);
- Contextual factors of the EtD, including priority of the problem, cost, cost effectiveness, impact on equity, acceptability and feasibility.

In terms of implications for future research, the adaptation process allowed us to highlight the scarcity of regional data related to rheumatoid arthritis when looking at contextual factors such as cost, cost effectiveness and values and preferences. This should encourage researchers to address these gaps through conducting primary studies. This will allow the adaptation process to be better contextualized and the data on which we rely on to be more systematic and evidence based rather than solely relying on the panels knowledge and opinions.

Schünemann et al. described a vision of guideline development through an “international collaboration with common aims and free of proprietary influences” [21]. Several of the concepts highlighted in the statement are highly relevant to a successful adaptation efforts. (1) Globalizing the evidence through a standardized database of existing evidence and gaps to serve as a shared resource for participating organizations; organizations interested in adapting guidelines could tap into this database to identify relevant source evidence summaries and evidence to decision frameworks. (2) Undertaking collaborative evidence reviews relevant to recommendation questions; while the source guideline working group develop the base evidence summaries based on high certainty systematic reviews, the adaptation working groups could contribute to their subsequent update. (3) Maintaining a collaborative network of organizations with common interests. This would allow a more efficient and effective coordination of efforts and allocation of resources [21].

## Conclusion

It is important for major guideline developers interested in others building on their guidelines, to develop them in a manner that facilitates the later adaptation process. This includes making explicit the methods used in the evidence synthesis, evidence grading and recommendation development processes to allow reproducibility. It also includes making the products of those processes available to other groups (search strategies, data files, grade tables). Finally, making explicit the judgments and underlying justifications on the EtD factors would make it easier to other groups to make their own judgments.

## Abbreviations

ACR: American College of Rheumatology
AUB: American University of Beirut
COI: Conflict of Interest
EMR: Eastern Mediterranean Region
EtD: Evidence to Decision
GDT: guideline development tool
MERAC: The Middle East Rheumatoid Arthritis Consortium
RA: Rheumatoid Arthritis
SoF: Summary of findings
WHO: World Health Organization

## References

[1] Steinberg E, Greenfield S, Mancher M, Wolman DM, Graham R. Clinical practice guidelines we can trust. Washington, DC: National Academies Press; 2011. 24983061

[2] World Health Organization (WHO) (2014). WHO handbook for guideline development.

[3] Fervers B, Burgers JS, Voellinger R (2011). Guideline adaptation: an approach to enhance efficiency in guideline development and improve utilisation. BMJ Qual Saf.

[4] Harrison MB, Légaré F, Graham ID (2010). Adapting clinical practice guidelines to local context and assessing barriers to their use. Can Med Assoc J.

[5] Darzi A, Abou-Jaoude EA, Agarwal A, et al. Frameworks for adaptation of health guidelines: a methodological survey. J Clin Epidemiol. 2017. doi:10.1016/j.jclinepi.2017.01.016. 10.1016/j.jclinepi.2017.01.016 28412463

[6] Alonso-Coello P, Schunemann HJ, Moberg J (2016). GRADE Evidence to Decision (EtD) frameworks: a systematic and transparent approach to making well informed healthcare choices. 1: Introduction. BMJ.

[7] Schunemann HJ, Wiercioch W, Brozek J, et al. GRADE evidence to decision frameworks for adoption, adaptation and de novo development of trustworthy recommendations: GRADE-ADOLOPMENT. J Clin Epidemiol. 2016; doi:10.1016/j.jclinepi.2016.09.009. 10.1016/j.jclinepi.2016.09.009 27713072

[8] Singh JA, Saag KG, Bridges SL (2016). 2015 American College of Rheumatology guideline for the treatment of rheumatoid arthritis. Arthritis Rheumatol.

[9] Schünemann HJ, Wiercioch W, Etxeandia I (2014). Guidelines 2.0: Systematic development of a comprehensive checklist for a successful guideline enterprise. Can Med Assoc J.

[10] AMSTAR (2007). Assessing the methodological quality of systematic reviews.

[11] GRADEpro GDT (2017). GRADEpro guideline development too.

[12] Guyatt G, Oxman AD, Akl EA (2011). GRADE guidelines: 1. Introduction-GRADE evidence profiles and summary of findings tables. J Clin Epidemiol.

[13] Alonso-Coello P, Oxman AD, Moberg J (2016). GRADE Evidence to Decision (EtD) frameworks: a systematic and transparent approach to making well informed healthcare choices. 2: Clinical practice guidelines. BMJ.

[14] Stoffer MA, Schoels MM, Smolen JS (2016). Evidence for treating rheumatoid arthritis to target: results of a systematic literature search update. Ann Rheum Dis.

[15] Gaujoux-Viala C, Nam J, Ramiro S (2014). Efficacy of conventional synthetic disease-modifying antirheumatic drugs, glucocorticoids and tofacitinib: a systematic literature review informing the 2013 update of the EULAR recommendations for management of rheumatoid arthritis. Ann Rheum Dis.

[16] Menon N, Kothari S, Gogna A (2014). Comparison of intra-articular glucocorticoid injections with DMARDs versus DMARDs alone in rheumatoid arthritis. J Assoc Physicians India.

[17] De Cock D, Vanderschueren G, Meyfroidt S (2014). Two-year clinical and radiologic follow-up of early RA patients treated with initial step up monotherapy or initial step down therapy with glucocorticoids, followed by a tight control approach: lessons from a cohort study in daily practice. Clin Rheumatol.

[18] Verschueren P, De Cock D, Corluy L (2015). Methotrexate in combination with other DMARDs is not superior to methotrexate alone for remission induction with moderate-to-high-dose glucocorticoid bridging in early rheumatoid arthritis after 16 weeks of treatment: the CareRA trial. Ann Rheum Dis.

[19] Scott DL, Ibrahim F, Farewell V (2015). Tumour necrosis factor inhibitors versus combination intensive therapy with conventional disease modifying anti-rheumatic drugs in established rheumatoid arthritis: TACIT non-inferiority randomised controlled trial. BMJ.

[20] Heimans L, Wevers-de Boer K, Visser K (2014). A two-step treatment strategy trial in patients with early arthritis aimed at achieving remission: the IMPROVED study. Ann Rheum Dis.

[21] Schunemann HJ, Woodhead M, Anzueto A (2009). A vision statement on guideline development for respiratory disease: the example of COPD. Lancet.

