# Correction to: Adaptation of the 2015 American College of Rheumatology treatment guideline for rheumatoid arthritis for the Eastern Mediterranean Region: an exemplar of the GRADE Adolopment

**DOI:** 10.1186/s12955-017-0791-9

**Published:** 2017-10-26

**Authors:** Andrea Darzi, Manale Harfouche, Thurayya Arayssi, Samar Alemadi, Khalid A. Alnaqbi, Humeira Badsha, Farida Al Balushi, Bassel Elzorkany, Hussein Halabi, Mohammed Hamoudeh, Wissam Hazer, Basel Masri, Mohammed A. Omair, Imad Uthman, Nelly Ziade, Jasvinder A. Singh, Robin Christiansen, Peter Tugwell, Holger J. Schünemann, Elie A. Akl

**Affiliations:** 1 AUB GRADE Center, Clinical Research Institute, American University of Beirut, PO Box 11-0236, Riad El Solh, Beirut, 1107 2020 Lebanon; 2 Weill Cornell Medicine-Qatar- Department of Internal Medicine, Doha, Qatar; 3 Department of Rheumatology, Medical Institute, Al Ain Hospital, Al Ain, United Arab Emirates; 4 Dr. Humeira Badsha Medical Center, Rheumatologist City Hospital, Rheumatologist Neurospinal Hospital, Dubai, United Arab Emirates; 5 Ministry of Health, Muscat Governorate, Oman; 6 Department of Rheumatology, Cairo University, Giza, Egypt; 7 Rheumatology Division, Department of Internal Medicine, King Faisal Specialist Hospital and Research Center, Jeddah, Saudi Arabia; 8 Hamad Medical Corporation, Doha, Qatar; 9 Aspetar, Doha, Qatar; 10 Jordan Hospital, Amman, Jordan; 11 Division of Rheumatology, Department of Medicine, King Saud University, Riyadh, Saudi Arabia; 12 American University of Beirut, Beirut, Lebanon; 13 Faculty of Medicine, Univeristé Saint Joseph, Beirut, Lebanon; 14 Medicine Service and Center for Surgical Medical Acute care Research and Transitions, VA Medical Center, 510, 20th Street South, Birmingham, AL FOT 805B USA; 15 Department of Medicine at School of Medicine, and Division of Epidemiology at School of Public Health, University of Alabama, 1720 Second Ave. South, Birmingham, AL 35294-0022 USA; 16 Musculoskeletal Statistics Unit, The Parker Institute, Bispebjerg and Frederiksberg Hospital, Copenhagen, Denmark; 17 Department of Medicine, University of Ottawa, Ottawa, Canada; 18 Department of Medicine, McMaster University, Hamilton, ON Canada; 19 Department of Health Research Methods, Evidence, and Impact (HE&I), McMaster University, Hamilton, ON Canada; 20 Department of Internal Medicine, American University of Beirut, PO Box 11-0236, Riad El Solh, Beirut, 1107 2020 Lebanon

## Correction

The original article [1] contained an error whereby the co-author, Khalid A. Alnaqbi’s name was spelt incorrectly.

This error has now been corrected.
